# Aging Differentially Affects Multiple Aspects of Vesicle Fusion Kinetics

**DOI:** 10.1371/journal.pone.0027820

**Published:** 2011-11-18

**Authors:** Mark P. Zanin, Lucy Phillips, Kimberly D. Mackenzie, Damien J. Keating

**Affiliations:** Department of Human Physiology and Centre for Neuroscience, Flinders University, Adelaide, Australia; The University of Queensland, Australia

## Abstract

How fusion pore formation during exocytosis affects the subsequent release of vesicle contents remains incompletely understood. It is unclear if the amount released per vesicle is dependent upon the nature of the developing fusion pore and whether full fusion and transient kiss and run exocytosis are regulated by similar mechanisms. We hypothesise that if consistent relationships exist between these aspects of exocytosis then they will remain constant across any age. Using amperometry in mouse chromaffin cells we measured catecholamine efflux during single exocytotic events at P0, 1 month and 6 months. At all ages we observed full fusion (amperometric spike only), full fusion preceded by fusion pore flickering (pre-spike foot (PSF) signal followed by a spike) and pure “kiss and run” exocytosis (represented by stand alone foot (SAF) signals). We observe age-associated increases in the size of all 3 modes of fusion but these increases occur at different ages. The release probability of PSF signals or full spikes alone doesn't alter across any age in comparison with an age-dependent increase in the incidence of “kiss and run” type events. However, the most striking changes we observe are age-associated changes in the relationship between vesicle size and the membrane bending energy required for exocytosis. Our data illustrates that vesicle size does not regulate release probability, as has been suggested, that membrane elasticity or flexural rigidity change with age and that the mechanisms controlling full fusion may differ from those controlling “kiss and run” fusion.

## Introduction

The major mechanism underlying neurotransmitter and hormone release is exocytosis, the formation of a fusion pore between vesicle and plasma membranes resulting in release of vesicular contents into the extracellular space. Carbon-fibre amperometry has provided a significant proportion of our current understanding of exocytosis, with several different types of exocytosis events detectable using this method. These events can be classified as spikes, spikes with pre-spike foot (PSF) signals and stand-alone foot (SAF) signals. Spikes result from the rapid stabilization of the fusion pore followed by substantial, possibly total, release of vesicle contents. PSF signals represent transient release through the “flickering” of an unstable, possibly proteinacious, fusion pore prior to pore stabilization [1], [2]. Less commonly, a foot signal without a proceeding spike is observed. This defines a SAF signal and represents pure ‘kiss and run’ type fusion, where a fraction of vesicle content is released through the unstable fusion pore before pore closure or collapse. What dictates the occurrence of each form of exocytosis remains an area of ongoing investigation. Previous amperometric studies indicate that these different events may be interdependent. PSF signal size has been correlated with the amount released per vesicle [3] while total vesicle content may positively influence release probability [4] and the probability of a PSF signal occurring [5]. How these factors relate to each other and the exact nature of their interdependencies has not been fully characterised and requires further investigation.

Understanding the stringent regulation of exocytosis is important in health and disease as it controls the release of molecules which regulate functions including cognition, synaptic transmission and blood glucose levels. A loss of control of these processes can underlie pathological outcomes including hypertension, psychiatric and neurodegenerative disorders and diabetes. Aging is also an important factor in these disorders. The expression of several proteins involved in exocytosis, including SNAP-25, VAMP and Munc-18, decrease with aging [6], [7]. Vesicle size within rat chromaffin cells also increases with age [8] and vesicle size is thought to affect the fusion of vesicles with the cell membrane [5], [9] and the probability of neurotransmitter release [4]. Recent analytical advances illustrate a linear relationship between PSF signal duration, vesicle size and the membrane bending properties associated with the transition from reversible to irreversible fusion pore states [1].

In the current study we used carbon-fiber amperometry to understand the effects of aging on exocytosis in adrenal chromaffin cells obtained from newborn (P0), 1 month old and 6 month old mice. The total amount released per vesicle was lower at P0 compared to 1 and 6 months and similar, but not identical, changes were seen in PSF signal parameters. Age-related changes in the number of amperometric spikes or frequency of PSF signals were not observed and this differed to the SAF signal frequency, which increased with age. Most strikingly we illustrate significant age-associated changes in the membrane bending properties of these cells. Thus we illustrate novel age-related differences in the occurrence of full fusion versus transient “kiss and run” type fusion and identify significant changes in the energy requirements for the transition from fusion pore formation to full fusion with age.

## Results

### The probability of “kiss and run”, but not full fusion, changes with aging

**Figure 1:**
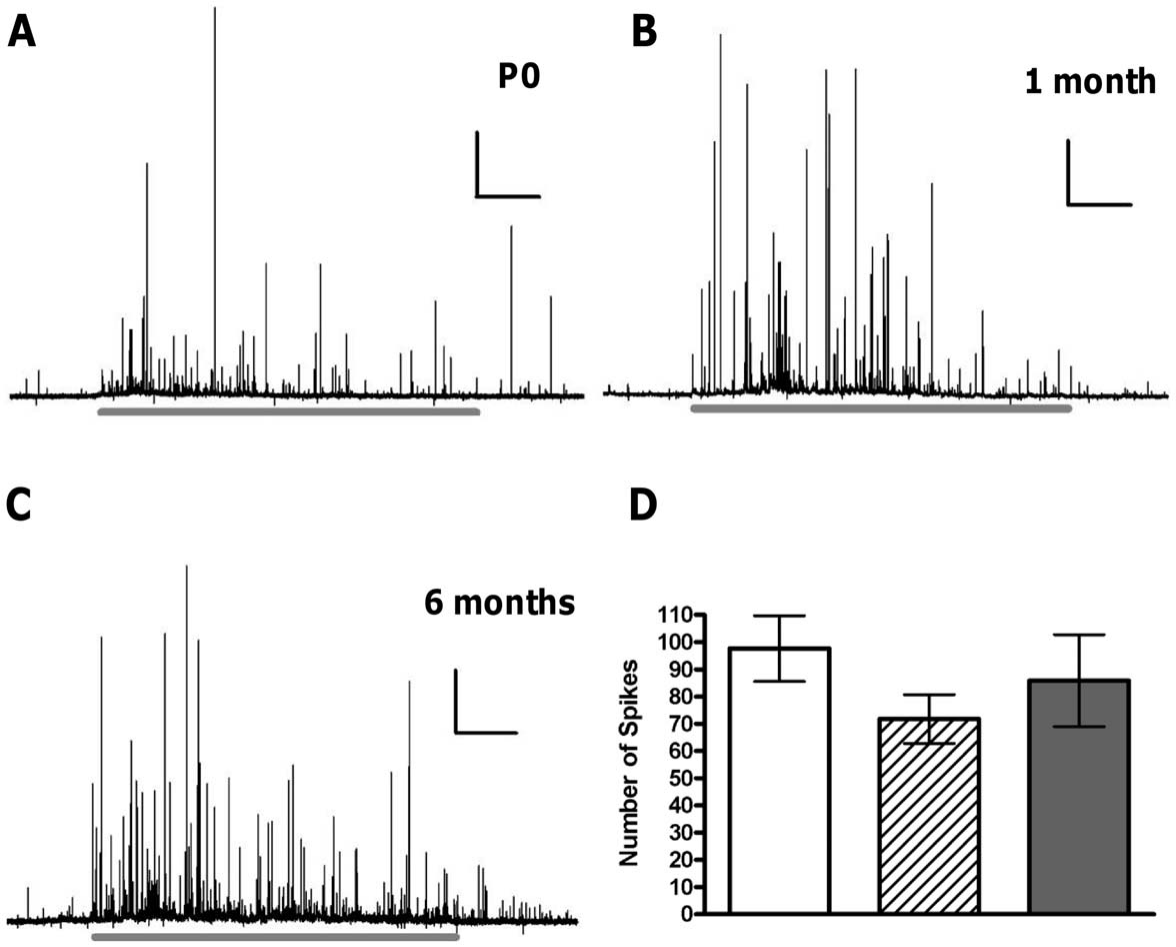
Aging does not affect the number of vesicles undergoing full fusion. Representative amperometric traces show the 60 second stimulation period (grey line below trace) in cells from (A) P0, (B) 1 month and (C) 6 months. The average number of events for each cell (D) illustrates the number of exocytotic events does not change with age. n = 14, 15 and 16 for P0 (white bar), 1 month (striped bar) and 6 months (grey bar), respectively. Scale bars in (A–C) represent 10sec and 100 pA.

**Figure 2:**
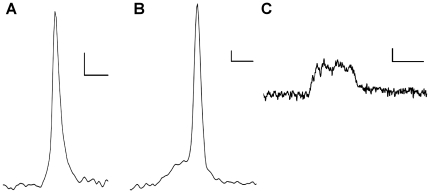
Different types of fusion as observed using carbon fibre amperometry. A full fusion event without a pre-spike foot signal (A), a full fusion event with a pre-spike foot signal (B) and a kiss and run event represented as a stand-alone foot signal (C) are shown. Scale bars represent 2 ms and 10 pA.

We stimulated cells for 60 seconds and measured the total number of exocytotic events during that time in all 3 age groups investigated. Each single spike in these traces represents an individual vesicle undergoing exocytosis and releasing its catecholaminergic contents. We observed no significant alteration in the number of events occurring in any group, with 97.6±12.0 events in P0 cells, 78.6±8.6 at 1 month and 85.8±16.9 at 6 months (Figure 1). Increasing the temporal resolution of these events illustrates that 3 major types of fusion occur. Spikes without a PSF signal are observed, representing fusion without flickering of the developing fusion pore (Figure 2A). Full amperometric spikes with a PSF signal represent full fusion preceded by a prolonged unstable flickering of the fusion pore (Figure 2B). We also observed SAF signals which represent flickering of the fusion pore followed by rapid pore closure or collapse before the full fusion pore has developed (Figure 2C). These SAF signals represent what is commonly termed as “kiss and run” type fusion.

**Figure 3:**
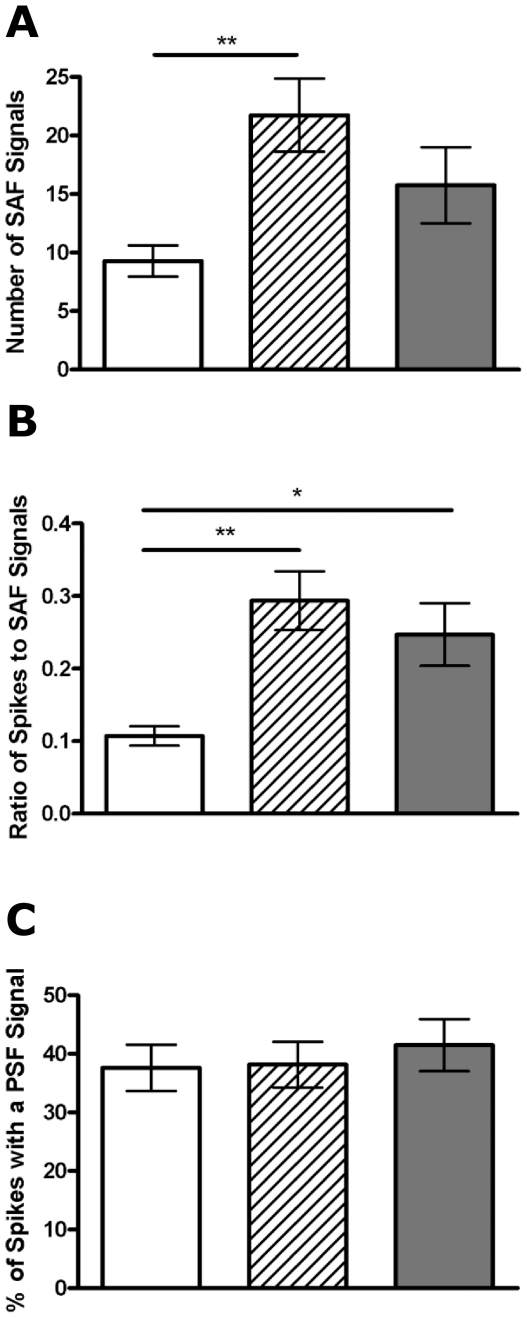
Aging affects the incidence of pre-spike foot signals and stand-alone foot signal events differently. The number of stand-alone foot (SAF) signals observed increases with age (A) as does the ratio of SAF signals to full spikes (B), while the percentage of spike displaying a pre-spike foot (PSF) signal is maintained across all ages studied (C). n = 14, 15 and 16 cells for P0 (white bar), 1 month (striped bar) and 6 month (grey bar), respectively. *, *p*<0.05, **, *p*<0.01, calculated by Mann-Whitney U tests.

We then measured the occurrence of the SAF and PSF signals. If these events simply involve identical vesicles that, at the point of transient fusion pore opening, either do or do not develop a large, irreversible, stable fusion pore, then we assume that the frequency of these signals should be similarly affected by aging. We find that the number of SAF signals per cell increases with age significantly between P0 (9.3±1.3 events) and 1 month (21.7±3.2, *p*<0.01) and at 6 months 15.7±3.2 SAF signals per cell were observed (Figure 3A). The ratio of full fusion vs. kiss and run fusion is significantly lower also at P0 (0.1±0.01) compared to either 1 month (0.29±0.04, *p*<0.01) or 6 months (0.25±0.04, *p*<0.05, Figure 3B). However the frequency of PSF signals observed did not change at any age (Figure 3C).

### Vesicle size increases with aging

**Figure 4:**
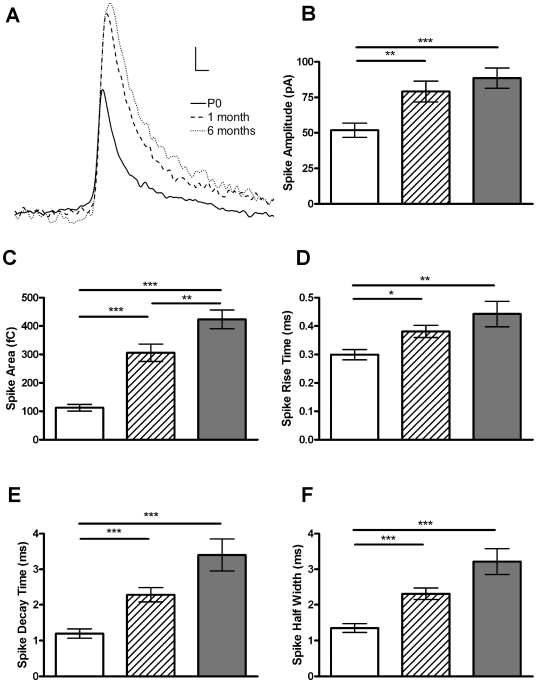
Aging alters the kinetics of full fusion. (A) Examples of a typical amperometric spike at P0, 1 month and 6 months. Increases in spike amplitude (B), area (C), rise time (D), decay time (E) and half-width (F) are all evident, indicating that release kinetics and the amount released from each vesicle increases with age. Graphs represent the mean of each cells median value ± SEM. *, *p*<0.05; **, *p*<0.01; ***, *p*<0.001, calculated by Mann-Whitney U tests. n = 14, 15 and 16 for P0 (white bar), 1 month (striped bar) and 6 month (grey bar), respectively. Scale bars in (A) represent 10 ms and 10 pA.

We next measured the kinetics of full fusion events in all 3 age groups (Table 1). Representative spikes from each age are shown (Figure 4A). All aspects of fusion kinetics were significantly lower at P0 compared to other ages and no differences were seen between 1 and 6 months with the exception of spike area, which was significantly lower at 1 month compared with 6 months (*p*<0.01, Figure 4B–F). We consequently observe significant age-related increases in the total amount of catecholamine released per cell. The total amount released per cell in P0 cells (8.4±1.6 nC) was significantly less than at either 1 (19.6±4.5 nC, *p*<0.05) or 6 (26.5±.0 nC, *p*<0.01) months.

**Table 1:** Effect of aging on single amperometric events.

	Number of Events	Spike Amplitude (pA)	Spike Charge (pC)	Spike Rise Time (ms)	Spike Decay Time (ms)	Spike Half-width (ms)	Number of cells
P0	97.6±12.0	51.8±5.0	112.3±11.6	0.29±0.02	1.19±0.13	1.3±0.1	14
1 month	71.7±8.6	79.1±7.4	306.1±30.4	0.38±0.02	2.28±0.20	2.3±0.2	15
6 months	85.8±16.9	88.5±7.1	423.4±33.0	0.44±0.04	3.40±0.45	3.2±0.4	16

Values are averages of cell median values ± SEM.

### Characteristics of fusion pore development also change with age

**Figure 5:**
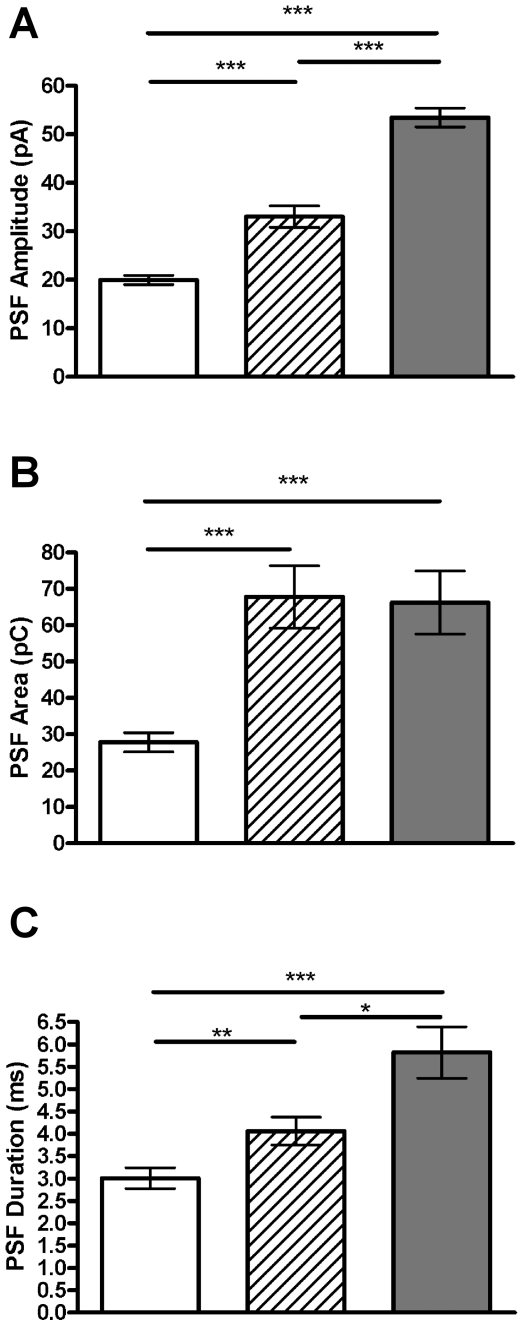
The kinetics of the transient fusion pore increase with age. The amplitude (A), area (B) and duration (C) of the pre-spike foot (PSF) signal change with aging. Graphs show means of all PSF signals at each age tested ± SEM. *, *p*<0.05; **, *p*<0.01; ***, *p*<0.001, calculated by Mann-Whitney U tests. n = 14, 15 and 16 cells and 466, 268, 599 events for P0 (white bar), 1 month (striped bar) and 6 month (grey bar), respectively.

We next investigated the changes in PSF signal parameters with age (Table 2). Similar to the effect of age on full spike kinetics, we also observe increases in PSF signal parameters with age. PSF signal area increases at 1 and 6 months compared to P0 (*p*<0.001, Figure 5B). Significant differences between all ages were observed for both PSF signal amplitude and duration (Figure 5A and C). These age-associated changes lead us to further investigate the interdependence between fusion pore formation, vesicle size and membrane elasticity.

**Table 2:** Effect of aging on pre-spike foot signals.

	Pre-Spike Foot Frequency (%)	Pre-Spike Foot Amplitude (pA)	Pre-Spike Foot Area (pC)	Pre-Spike Foot Duration (ms)	Number of cells
P0	37.6±3.9	19.9±0.9	27.8±2.6	3.0±0.2	14
1 month	40.1±3.3	16.2±1.1	33.0±2.2	4.1±0.3	15
6 months	41.5±4.4	53.5±1.9	66.2±8.7	5.8±0.6	16

Values are averages of all cells ± SEM.

### Aging affects the relationship between transient fusion pore opening and full fusion release kinetics

**Figure 6:**
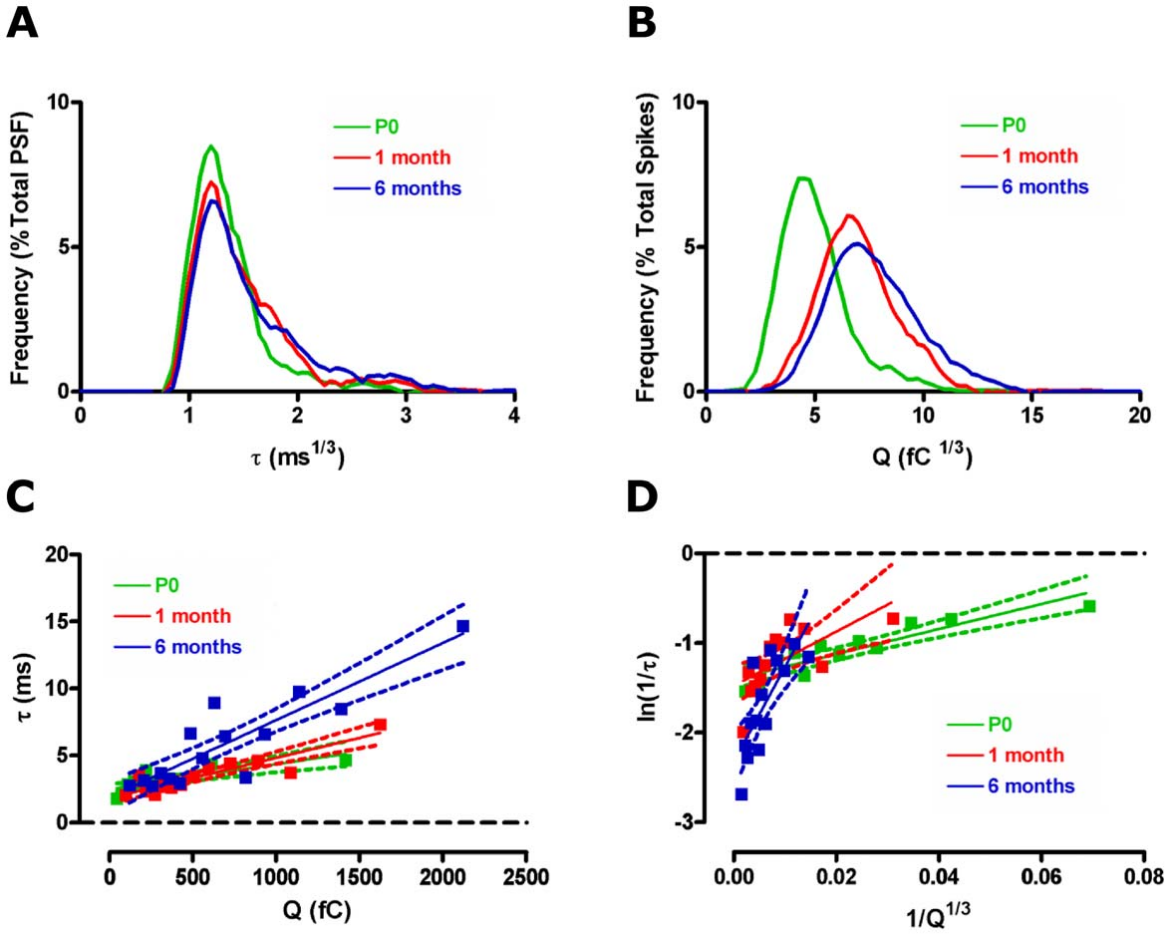
Aging differentially affects membrane bending properties during exocytosis. Frequency distribution of the cube root of pre-spike foot (PSF) signal duration (A) and spike area (B) illustrates the different ages at which these change. Correlations between PSF signal duration, τ, and spike area, Q (C) shows a linear relationship at all ages (P0: R^2^ = 0.64, *p*<0.01, 1 month: R^2^ = 0.82, *p*<0.0001, 6 months: R^2^ = 0.79, *p*<0.0001). Transforms of this data to ln(1/τ) vs 1/Q^1/3^ (D) also provides linear relationships at all ages (P0: R^2^ = 0.81, *p*<0.0001, 1 month: R^2^ = 0.44, *p*<0.05, 6 months: R^2^ = 0.59, *p*<0.01). The slope of this plot, representing membrane curvature [1], increases significantly with age. Green – P0, red – 1 month, blue – 6 months.

Recent investigations highlight the relationship between PSF signal duration and vesicle size, which provides insights into membrane curvature and membrane bending properties during exocytosis [1]. Spike area scales directly with vesicle size [1], [10], [11], which increases with age. We used histograms to illustrate the age-related changes observed in the frequency distribution of PSF signal duration, τ, and spike area, Q. As these data sets are non-parametrically distributed we first transformed them to create parametric data sets. Such frequency distribution plots (Figure 6A) illustrate that the age-related change in mean PSF signal duration is caused by a subset of longer PSF signals in the 1 and 6 month group compared to the P0 group. However when we observe the relative distribution of Q we see a clear population shift to the right in the 1 and 6 month age groups (Figure 6B), indicating a shift towards larger vesicle sizes at these ages.

Fusion pores formed by smaller vesicles dilate more rapidly than those formed by larger vesicles [1]. This is illustrated by binning Q and averaging the corresponding τ within each bin to produce linearly correlated plots. We observe this relationship between Q and τ at each age studied (r^2^ = 0.65 at P0, 0.82 at 1 month and 0.8 at 6 months) and see a difference in the slope with aging (Figure 6C). This slope is significantly less at P0 compared to both 1 and 6 months (*p*<0.05) and is less at 1 month compared to 6 months (*p*<0.05, Figure 6C). Given that reaction rates are generally exponential functions of energy we transformed the y axis to ln(1/τ). As a vesicle of radius R_v_ has a membrane curvature of 1/R_v_, and as the volume of a vesicle scales with Q, the curvature of a spherical vesicle should vary when 1/Q^1/3^ [1]. Transforming the plots in Figure 6C to plots of ln(1/τ) vs. 1/Q^1/3^ maintained the linear relationships observed when correlating τ and Q. When we compare this relationship amongst different ages we observe stark changes in the slope (P0 = 13.9±2.1, 1 month  = 30.2±10.4 and 6 months  = 105.9±25.7, Figure 6D). These differences are significant between P0 and 6 months (*p*<0.05).

### SAF and PSF signals alter differently with age

**Figure 7:**
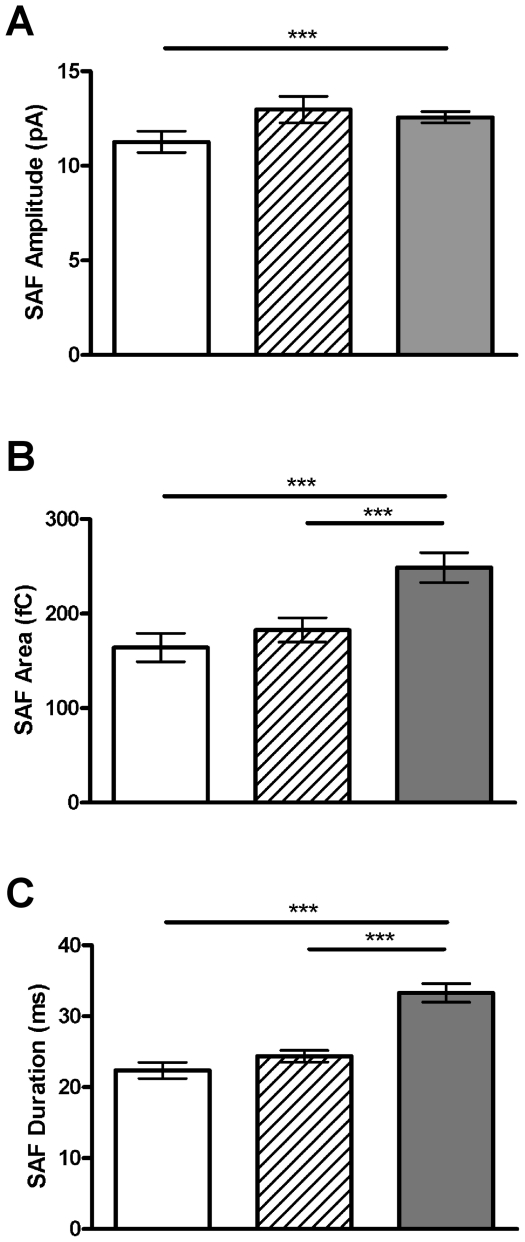
The kinetics of kiss and run fusion events changes with aging. The amplitude (A), area (B) and duration (C) of stand-alone foot (SAF) signals all change with aging. Graphs show means of all SAF signals at each age tested ± SEM. ***, *p*<0.001, calculated by Mann-Whitney U tests. n = 14, 15 and 16 cells and 130, 273 and 252 events for P0 (white bar), 1 month (striped bar) and 6 month (grey bar), respectively.

We also studied the characteristics of SAF signals in order to see how the kinetics of kiss and run exocytosis changes with age (Table 3). SAF signal amplitude is significantly lower at P0 compared to 6 months (*p*<0.001, Figure 7A). Both SAF signal area and duration are significantly less at P0 compared to 1 month or 6 months (*p*<0.001, Figure 7B and C). This is in contrast to the changes observed in the area and duration of the PSF signal, where significant differences are observed between all age groups (Figure 5).

**Table 3:** Effect of aging on ‘kiss and run’ events.

	Number of Events	Amplitude (pA)	Charge (pC)	Duration (ms)	Number of cells
P0	9.3±1.3	11.3±0.56	164.1±15.1	22.3±1.13	14
1 month	21.7±3.2	11.4±0.79	154.9±12.4	23.7±0.80	15
6 months	15.7±3.2	12.6±0.30	248.8±15.70	33.3±1.30	16

Values are averages of all cells ± SEM.

### Alterations in Ca^2+^ entry do not explain aging-related changes in exocytosis

**Figure 8:**
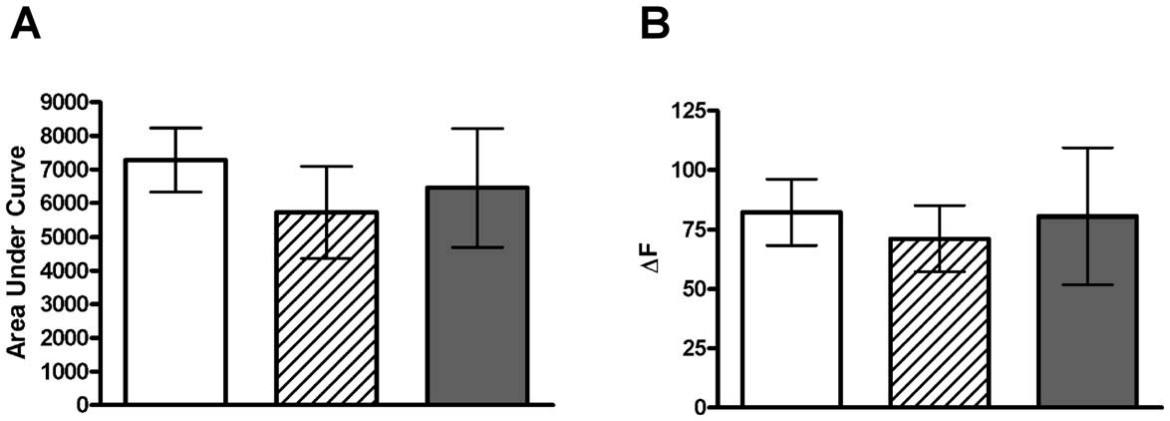
Ca^2+^ entry is not altered with age in mouse chromaffin cells. Cells from each age were loaded with the Ca^2+^-sensitive dye, Fluo-4, and stimulated for 60 sec with 70 mM K^+^ solution. The increase in fluorescence reflects Ca^2+^ entry and we find that the total increase in fluorescence (area under the curve; A) and the peak fluorescence change (ΔF; B) are unchanged at any age. Data is mean ± SEM, significance tested using one-way ANOVA. n = 16, 9 and 8 cells from P0 (white bar), 1 month (striped bar) and 6 month (grey bar), respectively. Cells were obtained from >3 mice in each age group.

As we observe clear age-related changes in multiple facets of exocytosis, we measured whether alterations in Ca^2+^ entry or Ca^2+^ handling are altered with age. Cells from each age were loaded with the Ca^2+^-sensitive dye, Fluo-4, and stimulated in an identical fashion to that which occurred for amperometry experiments. To gauge the Ca^2+^ handling properties of these cells we measured the area under the curve upon stimulation-induced increases in cell fluorescence (Figure 8A). We observe no difference between the three groups. We also observe no difference between groups in terms of the maximal fluorescence (ΔF) change observed (Figure 8B). We therefore conclude that differences in Ca^2+^ entry and Ca^2+^ handling do not underlie the age-associated changes we observe in various aspects of exocytosis.

## Discussion

We provide new insight into the changing nature of exocytosis with age. The number of full fusion events and the frequency of PSF signals are not affected by age as opposed to the number of SAF signals, representing pure kiss and run events, which increase with age. Additionally, the kinetics of full fusion, PSF and SAF signals increase with age. The fact that the changes in kinetics of these various fusion modes alter differently with aging suggests different regulatory mechanisms may underlie full fusion and kiss and run exocytosis. When we compare PSF signal duration with spike area we observe a linear relationship. However, the slope of this relationship is significantly increased with age, inferring strong changes in membrane bending properties over time. Our findings are relevant to the understanding of the basic mechanisms of exocytosis in all excitable cell types, including neurons, in which kiss and run exocytosis, fusion pore flickering and full fusion exocytosis are all known to occur [12], [13], [14].

### Kiss and run and full fusion probabilities are differentially affected by age

One of our first observations in this study was that the number of full fusion events is not affected by aging as observed in a previous comparison of maternal and embryonic rat chromaffin cells [15]. We similarly find no age-dependence of the probability of a PSF signal but observe a clear increase in both the number of SAF signals and the ratio of kiss and run to full fusion events with age. A variety of factors are known to regulate kiss and run type fusion, including synaptotagmin 1 and 4 and forskolin [16], Ca^2+^ [17] and cholesterol [18]. It will be of interest to know whether changes in any of these factors dictate this age-dependent increase specifically in kiss and run fusion. Our results indicate that changes in Ca^2+^ entry are unlikely to be responsible for the alterations we observe. This also fits with the lack of change in the number of full fusion events with age.

One possible conclusion from our observations is that the control mechanisms regulating the occurrence of transient and full fusion exocytosis may differ. This is based on the simplistic assumption that if the mechanisms underlying these two fusion modes were the same then we would not have seen different effects of age on their respective frequencies. However this conclusion is largely speculative at this stage. What these different controlling factors are and how they change with aging will be an area of further investigation.

### Membrane bending properties alter with aging

We observe significantly less release per vesicle (spike charge) at P0 compared to other ages, similar to comparisons made between maternal and embryonic chromaffin cells [15]. Spike charge is directly correlated with vesicle size in chromaffin cells [1], [19] as these vesicles maintain a constant internal catecholamine concentration [10]. Therefore spike charge is a direct indicator of vesicle size and age-related increases in vesicle size have been previously documented in chromaffin cells [8]. Recent experiments have highlighted the linear relationship between fusion pore duration, which represents an estimate of the stability of the initial fusion pore, and vesicle size, which is represented by spike charge [1]. We observe the same relationship in our experiments and illustrate that the slope of this relationship changes significantly with age. Transforming this correlation to plots of ln(1/τ) versus 1/Q^1/3^ allowed us to compare elasticity of the fusing membranes with age. The increases with age that we observe in the slope of this relationship are not simply caused by the increasing vesicle size with age, as vesicle size changes only offset the intercept of these correlations and not the slope [1]. Rather, the increase in slope with age most likely reflects alterations in either membrane curvature properties or increasing membrane flexural rigidity [1], both of which will affect the slope of such relationships. Another, potentially more straightforward, way to interpret these changes with age is that, when comparing similar sized vesicles across the 3 ages, more energy is required for full fusion to occur in older cells.

Precisely what underlies these changes remains unknown although alterations in the lipid composition of the membrane bilayers involved are strong candidates given lipid perturbations alter the slope of the ln(1/τ) vs. 1/Q^1/3^ relationship [1]. One candidate lipid is cholesterol, which imparts high negative curvature on membranes, which correlates with its facilitation of membrane fusion [20], [21]. Cholesterol levels are decreased in the aging brain and in hippocampal neurons aged *in vitro* [22], [23], [24]. However the lowering of cholesterol in the cytoplasmic leaflet of chromaffin cells shortens τ and reduces the proportion of SAF signals, whereas an increase in internal cholesterol has opposite effects [18]. Additionally, cellular cholesterol levels do not alter Q but have a positive influence on the slope of the relationship between τ and Q [18]. We conclude from this that either cholesterol levels increase with age in mouse chromaffin cells or that altered cholesterol levels do not underlie the age-associated changes in exocytosis we observe. A full analysis of the lipid composition of vesicle and plasma membranes may be needed in order to identify whether these factors are altered in aging chromaffin cells.

### Kiss and run fusion and full fusion are differentially controlled

A simplistic explanation of the process of exocytosis is that the vesicle and plasma membranes initiate the formation of a fusion pore which either transforms into a stable fusion pore in full fusion or collapses, resulting in kiss and run exocytosis. Such an explanation might assume that, prior to fusion, a vesicle has the potential to undergo either type of fusion. If true, then factors that affect exocytosis with age would be assumed to affect both types of fusion equally. In our experiments, the probabilities of observing full spikes or PSF signals remain constant with age while SAF signal probability and the SAF to full spike ratio increase with age. This result suggests alternate control mechanisms controlling transient and full fusion, the existence of vesicle heterogeneity in terms of their protein and lipid components, or heterogeneity of the protein and lipid components at sites of fusion on the plasma membrane.

If the energy required to undergo the transition from an early, potentially proteinaceous, fusion pore to a larger, more stable and potentially lipidic fusion pore is not adequate, the result may be the failure of such a transition occurring (ie; kiss and run fusion). Our finding that membrane bending energy is increased with age is a potential explanation as to why we observe more kiss and run exocytosis with age. Additionally, the mean duration of the SAF signal (∼22.3 – 33.2 ms) is vastly longer than that of the PSF signal (∼3.1 – 5.8 ms). Kiss and run events and full fusion events may therefore involve vesicle or plasma membrane properties which are inherently different from each other, with these differences dictating the type of exocytosis that will occur.

Whilst the reasons for these changes are unknown, factors that may play a role include vesicle-associated proteins, accessory proteins or the lipid composition of the interacting membranes. We have already described the potential effect of altering membrane cholesterol on fusion kinetics but the altered expression of various proteins may also play a role. A deletion mutant of SNAP-25 which effects C-terminal zippering results in longer PSF signal duration and reduced pore conductance [25] while guanine nucleotide exchange activity regulates both PSF and SAF signal frequency [26]. Different regions of synaptotagmin also effect fusion pore stability [27] and alter kiss and run probability [16]. The factors underlying the changes we observe are beyond the scope of our present study.

### Potential physiological outcomes for the different aging-related changes in pore formation and vesicle release

While our findings provide new insight into understanding the control of the fusion pore and the distal steps of exocytosis, they also have implications which are relevant to other areas of research. Chromaffin cells are the major source of circulating catecholamines. Modulation of fusion pore kinetics in these cells will have direct effects on the amount of catecholamine released in times of physiological stress. Our results show a significant increase in total catecholamine released per cell with aging, in line with the age-associated increases in circulating catecholamines observed in both rodents [28] and humans [29]. A surge of circulating catecholamines can have differential effects depending upon the developmental stage. In the fetus, these changes include decreased heart rate, a rise in fetal arterial pressure and dramatic increases in blood flow to the heart, brain and adrenal glands with a concomitant reduction in blood flow to the kidneys, gut, liver, lung and extremities [30]. In mature animals this same response triggers increased heart rate and changes in blood vessel diameter and air passage dilation as part of the “flight or fight” response. Adrenal chromaffin cells also release substantial amounts of biologically active peptides including PACAP and VIP. Recent evidence has clearly illustrated that modulation of fusion pore opening in chromaffin cells directly affects the release of such peptides [31].

In summary, we have illustrated previously uncharacterised age-dependent changes in the kinetics associated with fusion pore formation, vesicle release and kiss and run exocytosis. We also observe an effect of aging on the probability of kiss and run events but not on full fusion events. Our analysis indicates that membrane bending capacity or membrane flexural rigidity may possibly change with aging in chromaffin cells and that this may underlie the increased incidence of kiss and run type fusion with age. It will be important to determine the mechanisms underlying for these differences and what significance these have to our understanding of exocytosis regulation.

## Materials and Methods

### Ethics statement

C57Bl/6J mice were used for all experiments and all animals were killed in accordance with the Flinders University animal ethics committee. The Flinders University animal ethics committee approved the use of these animals in this study (approval numbers 620/06(a) and 715/09).

### Chromaffin cell culture

P0 mice were killed by decapitation whilst 1 month and 6 month old mice were killed by overdose with isofluorane. Adrenal glands were removed from dead mice and the adrenal medulla was dissected from the gland in cold Locke's Buffer (154 mM NaCl, 5.6 mM KCl, 3.6 mM NaHCO_3_, 5.6 mM glucose, 5.0 mM HEPES, pH 7.4) and incubated with collagenase type A (3 mg/mL in Locke's Buffer) (Roche, Germany) in a shaking water bath at 37°C. The collagenase was then diluted in cold Locke's Buffer and cells were pelleted and resuspended in supplemented DMEM (Dulbecco's modified Eagle's medium supplemented with 10% (v/v) heat inactivated fetal calf serum, 100 units/mL penicillin and 100 mg/mL streptomycin (Invitrogen, Carlsbad, CA, USA) and filtered through a nylon mesh. Cells were pelleted, resuspended in supplemented DMEM, plated on 35 mm tissue culture dishes and incubated at 37°C with 5% CO_2_. Cells were maintained in primary culture for 3 to 4 days prior to experiments.

### Amperometry

Catecholamine release from single chromaffin cells was measured using amperometry [32]. A carbon-fiber electrode (ProCFE, Dagan Corporation, USA) was placed on a chromaffin cell and +800 mV applied to the electrode under voltage clamp conditions. Current due to catecholamine oxidation was recorded using an EPC-9 amplifier and Pulse software (HEKA Electronic, Germany), sampled at 10 kHz and low-pass filtered at 1 kHz. For quantitative analysis files were converted to Axon Binary Files (ABF Utility, version 2.1, Synaptosoft, USA) and secretory spikes analysed (Mini Analysis, version 6.0.1, Synaptosoft, USA) for a period of 60 s from the start of stimulation. Each carbon fibre electrode was typically used no more than five recordings in order to avoid probe desensitisation. The standard bath solution contained 140 mM NaCl, 5 mM KCl, 2 mM CaCl_2_, 1 mM MgCl_2_, 5 mM D-glucose, 10 mM HEPES, pH 7.4. A solution containing a greater concentration of K^+^, used to stimulate cells, was identical in composition to the standard bath solution with 70 mM K^+^ replacing an equimolar amount of NaCl. All solutions were applied to cells using a gravity perfusion system, the outlet of which was placed within 500 µm of the cell being recorded. All experiments were carried out at 37°C using an in-line solution heater (Warner Instruments, USA).

### Data analysis

Amperometric spikes were selected for analysis of event frequency if spike amplitude exceeded 2.5 times the root-mean-squared noise of the baseline. Cells with fewer than 10 or more than 200 events within the 60 second stimulation period were excluded from analysis. For kinetic analysis of spikes and PSF signals, only those events that were not overlapping were included. Only PSF signals longer than 1 ms and above 2.5 times the root-mean-squared noise of the baseline were analyzed as foot signals. Rise time of each spike was calculated from the 50–90% rising phase in order to avoid skewing caused by PSF signals.

In analyzing spike kinetics, all spikes that met our threshold criteria were included in calculating the median values of each spike parameter for each cell. The averages of these median values were then used to compare each parameter between cell populations [33]. This was done to avoid errors associated with pooling large numbers of spikes from cells where there is a large cell to cell variability. Analysis of PSF and SAF signal kinetics was performed using pooled data from all recorded cells, as many recordings contained a low number of foot signals so that an adequate median value could not be obtained for each cell. PSF signal onset was defined when the signal exceeded the peak-to-peak noise of a 5 ms time segment, while the end of the PSF was defined as the inflection point between the PSF signal and the spike. PSF signal lifetime, τ, was taken as the intervening time interval. The total event area was taken as the integral from the PSF onset to the time where the spike current fell to 2.5 times the root-mean-squared noise of the baseline.

Non-parametrically distributed data sets were evaluated for statistical significance using the Mann–Whitney U test while parametrically distributed data sets were initially evaluated using One-way ANOVA followed by Tukey's post-hoc analysis. Statistical significance between slopes was designated based on non-overlapping 95% confidence intervals. *p*<0.05 was taken was the lowest level of statistical significance. All data presented are shown as mean ± SEM.

### Ca^2+^ imaging

Chromaffin cells were loaded with Fluo-4AM (Invitrogen, Australia, 5 µM) for 30 minutes at 37°C. Cells were stimulated for 60 seconds in Krebs buffer containing 70 mM K^+^ as described. Fluo-4 was excited using a Xenon light source (model LSLS-XL, Sutter Instruments, USA) and the emitted light filtered using an appropriate highpass filter. Images were obtained using a 10× water-immersion objective fitted to a CCD camera (Cascade II 512, Roper Photometrics, USA) on an upright microscope (Eclipse 50i, Nikon, Japan) at a 1 Hz rate. Analysis was performed with the Imaging Workbench software (version 6.01; Indec Biosystems, USA) for user-defined individual chromaffin cells.
